# A Template-Dependent Dislocation Mechanism Potentiates K65R Reverse Transcriptase Mutation Development in Subtype C Variants of HIV-1

**DOI:** 10.1371/journal.pone.0020208

**Published:** 2011-05-31

**Authors:** Dimitrios Coutsinos, Cédric F. Invernizzi, Daniela Moisi, Maureen Oliveira, Jorge L. Martinez-Cajas, Bluma G. Brenner, Mark A. Wainberg

**Affiliations:** 1 McGill University AIDS Center, Lady Davis Institute for Medical Research, Sir Mortimer B. Davis Jewish General Hospital, Montréal, Québec, Canada; 2 Departments of Microbiology and Immunology, McGill University, Montréal, Québec, Canada; 3 Department of Medicine, McGill University, Montréal, Québec, Canada; 4 Department of Medicine, Infectious Diseases, Queen's University, Kingston, Ontario, Canada; University of Pittsburgh, United States of America

## Abstract

Numerous studies have suggested that the K65R reverse transcriptase (RT) mutation develops more readily in subtype C than subtype B HIV-1. We recently showed that this discrepancy lies partly in the subtype C template coding sequence that predisposes RT to pause at the site of K65R mutagenesis. However, the mechanism underlying this observation and the elevated rates of K65R development remained unknown. Here, we report that DNA synthesis performed with subtype C templates consistently produced more K65R-containing transcripts than subtype B templates, regardless of the subtype-origin of the RT enzymes employed. These findings confirm that the mechanism involved is template-specific and RT-independent. In addition, a pattern of DNA synthesis characteristic of site-specific primer/template slippage and dislocation was only observed with the subtype C sequence. Analysis of RNA secondary structure suggested that the latter was unlikely to impact on K65R development between subtypes and that Streisinger strand slippage during DNA synthesis at the homopolymeric nucleotide stretch of the subtype C K65 region might occur, resulting in misalignment of the primer and template. Consequently, slippage would lead to a deletion of the middle adenine of codon K65 and the production of a -1 frameshift mutation, which upon dislocation and realignment of the primer and template, would lead to development of the K65R mutation. These findings provide additional mechanistic evidence for the facilitated development of the K65R mutation in subtype C HIV-1.

## Introduction

The human immunodeficiency virus type-1 (HIV-1) has evolved into four main groups, of which group M is responsible for the global HIV/AIDS pandemic and is further subdivided into multiple subtypes (A1, A2, B, C, D, F1, F2, G, H, J, and K) and circulating recombinant forms (CRFs) [1], [2], [3], [4], [5]. Currently, subtype B HIV-1 is responsible for approximately 12% of the global burden and is geographically concentrated in developed areas of the world, including North and South America, Europe, Australia and Japan. In contrast, subtype C HIV-1 is responsible for over 50% of new HIV infections and is geographically concentrated in sub-Saharan Africa, India and certain other developing countries [1]. The different subtypes differ by 10–12% in regard to nucleic acid sequences and 5–6% in amino acid sequences [6], [7], [8], [9]. The error-prone nature of the RT enzyme gives rise to viral diversity and is responsible for the emergence of drug resistance to antiretroviral therapy (ART) regimens that have been successful in curbing HIV-1 replication [10], [11], [12], [13].

The K65R mutation in HIV-1 RT is responsible for varying levels of reduced phenotypic susceptibility to most approved nucleotide and nucleoside reverse transcriptase inhibitors (N(t)RTIs) [14], [15], [16], [17], [18], [19], [20], [21]. K65R is also responsible for reductions in: viral fitness [22], [23], natural dNTP incorporation [24], N(t)RTI incorporation [25], [26], [27], [28], and N(t)RTI excision [29], [30]. Recent crystallographic studies suggest that the formation of a guanidinium plane between the arginines at positions 65 and 72 may be responsible for many of these observations [31]. In contrast, thymidine analogue mutations (TAMs) do not have the discriminatory properties of K65R and, as a result, are mutually exclusive with the latter and are rarely found on the same viral genome [29], [32], [33], [34].

We recently showed that subtype C HIV-1 developed the K65R mutation more readily than subtype B viruses in tissue culture [35]. In addition, numerous clinical studies have also demonstrated higher rates of K65R development ranging between 9 and 30% in subtype C-infected individuals who failed treatment [36], [37], [38], [39], [40], [41]. These rates are in marked contrast with subtype B HIV-1. K65R-associated treatment failures are uncommon and usually develop at rates between 0 and 3% [42], [43], [44], [45]. To explore this discrepancy, biochemical analysis of the RT enzymes derived from both subtypes were performed and showed that they are very similar; the subtype origin of RT cannot explain the discrepancies observed with respect to K65R development [46], [47].

**Figure 1:**
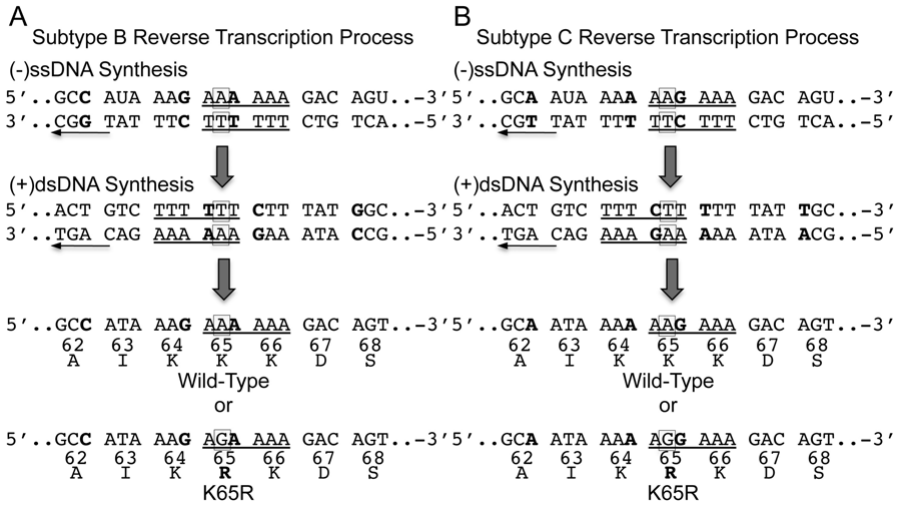
Sequence comparison of the RT region of the *pol* gene derived from subtype B and C HIV-1 spanning codons 62 to 68. (A) Outline of the reverse-transcription process in subtype B HIV-1 leading to either wild-type or K65R-containing transcripts. The K65R mutation results from an AAA-to-AGA mutation in subtype B HIV-1. (B) Outline of the reverse-transcription process in subtype C HIV-1 leading to either wild-type or K65R-containing transcripts. The K65R mutation results from an AAG-to-AGG mutation in subtype C HIV-1. The genomic sequences of the subtype B and C RT segments of *pol* spanning codons 62 to 68 are indicated. (−)ssDNA synthesis from the viral (+)ssRNA genome and (+)dsDNA synthesis from the (−)ssDNA intermediate are also depicted. Underlined are the homopolymeric nt stretches of both templates at their specific locations. In bold are the nt polymorphisms that exist between both subtypes in this region. Highlighted in a square is the base that, once mutated, gives rise to the K65R drug resistance mutation.

A single nucleotide (nt) mutation is required for both subtype B (AAA→AGA) (Figure 1A) and subtype C (AAG→AGG) (Figure 1B) viruses to develop the K65R mutation. Interestingly, subtype C viruses harbor a unique homopolymeric nucleic acid sequence at codons 64 and 65 of the RT segment of the *pol* gene which is also shared by subtypes F2, and H, and CRFs 07_BC, 08_BC and 31_BC. In addition, viruses from HIV-1 groups N and O as well as SIVcpz also share this particular subtype C sequence (Table 1). To investigate whether these polymorphisms in the viral templates might be involved in the preferential acquisition of K65R, we studied DNA synthesis from viral RNA and DNA templates derived from either subtype B or C HIV-1 [48]. The data showed that a strong pause site was present at the exact nt position responsible for K65R development during positive double-stranded DNA ((+)dsDNA) synthesis from the negative single-stranded DNA ((-)ssDNA) intermediate template. These findings were independent of the subtype-origin of the RT enzyme employed but were specific to the subtype-origin of the template. When two silent nt polymorphisms at codons 64 and 65 of the subtype C RT sequence were introduced into a wild-type NL4-3 virus of subtype B origin in cell culture, the resultant virus behaved like a subtype C virus in terms of K65R development [49]. Further investigation showed that the pausing reaction could not be alleviated by increasing individual nt concentrations and reaffirmed the template-specific and enzyme-independent nature of the findings [50]. Moreover, the pause site occurred at the end of a homopolymeric nt stretch; the synthesis of DNA from such regions is difficult for RT [51], [52], [53]. In addition, pause sites have been associated with strand transfer, recombination, misalignment and misincorporation events, all of which can lead to drug resistance [54], [55], [56], [57]. However, these data cannot confirm that the transcripts produced at the pause site contain K65R or some other mutational byproduct, or that the mutations produced may generate viable virus. Another recent report suggests that high-fidelity enzymes used in DNA amplification reactions as well as HIV-1 RT can also erroneously produce K65R [58]. These supplement our previous work but are not in contradiction with our findings. The current manuscript extends our previous results by providing an additional mechanistic basis for the hyper-presence of K65R in subtype C HIV-1.

**Table 1:** Sequence comparison of codons 64, 65 and 66 of the RT segment of *pol* of all main groups, subtypes and circulating recombinant forms of HIV-1 and of SIVcpz.

Name	Group/Subtype/CFR/Virus	Sequences of Codons 64, 65 and 66
*N	N	AAA AAG AAG
*O	O	AAA AAG AAG
A1	A1	AAG AAA AAA(G)
A2	A2	AAG AAA AAA
B	B	AAG AAA AAA
*C	C	AAA AAG AAA(G)
D	D	AAG AAA AAA
F1	F1	AAG AAA AAA
*F2	F2	AAG(A) AAA(G) AAA
G	G	AAG AAA AAA
*H	H	AAA AAG AAG
J	J	AAG AAA AAA
K	K	AAG AAA AAA
CRF01_AE	A, E	AAG AAA AAA
CRF02_AG	A, G	AAG AAA AAA
CRF03_AB	A, B	AAG AAA AAA
CRF04_cpx	A, G, H, K, U	AAG AAA AAG
CRF05_DF	D, F	AAG AAA AAA(G)
CRF06_cpx	A, G, J, K	AAG AAA AAA
*CRF07_BC	B, C	AAA AAG AAG
*CRF08_BC	B, C	AA(G)A AAG AAG
CRF10_CD	C, D	AAG AAA AAA
CRF11_cpx	A, CRF01, G, J	AAG AAA AAA(G)
CRF12_BF	B, F	AAG AAA AAA
CRF13_cpx	A, CRF01, G, J, U	AAG AAA AAA
CRF14_BG	B, G	AAG AAA AAG
CRF15_01B	CRF01, B	AAG AAA AAG
CRF16_A2D	A2, D	AAG AAA AAA
CRF17_BF	B, F	AAG AAA AAA
CRF18_cpx	A1, F, G, H, K, U	AAG AAA AAA
CRF19_cpx	A1, D, G	AAG AAA AAA
CRF20_BG	B, G	AAG AAG AAA
CRF21_A2D	A2, D	AAG AAA AAG
CRF22_01A1	CRF01, A1	AAG AAA AAA
CRF23_BG	B, G	AAG AAA AAA
CRF24_BG	B, G	AAG AAA AAA
CRF25_cpx	A, G, U	AAG AAA AAA(G)
CRF27_CPX	A, E, G, H, J, K, U	AA(G)G AAA AAA
CRF28_BF	B, F	AAG AAA AAA(G)
CRF29_BF	B, F	AAG AAA AAA
*CRF31_BC	B, C	AAA AAG AAG
CRF32_06A1	CRF06, A1	AAG AAA AAG
CRF33_01B	CRF01, B	AAG AAA AAA(G)
CRF34_01B	CRF01, B	AAG AAA AAG
CRF35_AD	A, D	AAG AAA AAA
CRF36_cpx	A, G, CRF01, CRF02	AA(G)G AAA AAA
CRF37_cpx	A, G, CRF01, CRF02, U	AAG AAA AAA
CRF39_BF	B, F	AAG AAA AAA
CRF40_BF	B, F	AAG AAA(G) AAA
CRF42_BF	B, F1	AAG AAA AAA
CRF43_02G	CRF02, G	AAG AAA AAA
*SIVcpz	SIVcpz	AAA AAG AAA

Asterisks mark the various groups, subtypes, CRFs and a related virus that share the unique coding sequence of subtype C HIV-1 RT at codons 64, 65 and 66 in RT.

We now show that the production of transcripts containing the mutagenic G nt at the middle position of codon 65 by HIV-1 RT is facilitated on subtype C templates and that template site-specific dislocation is the underlying mechanism responsible for the higher rates of K65R development. The findings are template-specific and enzyme-independent, are associated only with (+)dsDNA synthesis, and are not influenced by RNA secondary structure discrepancies between subtypes. We also evaluated the products of such dislocation that can yield both frameshift mutants in the pre-realignment phase and full-length K65R-containing transcripts in the post-realignment phase. These data provide mechanistic insight into K65R development in subtype C and are important in understanding the development of drug resistance in subtype C-infected patients.

## Materials and Methods

### Nucleic acid sequences

DNA templates and primers were purchased in desalted purity from Invitrogen (Carlsbad, CA, USA) or Integrated DNA Technologies (Coralville, IA, USA). Subtype B templates were derived from the NL4-3 virus and subtype C templates were derived from the MJ4 virus [48], [49], [50]. The subtype B-derived primer sequences used were: Subtype B-0MM-1: 5′-GGG GAC TTT GCC ATA AAG A-3′, Subtype B-1MM-2: 5′-GGG ACT TTG CCA TAA AGA G-3′, Subtype B-1MM-2C: 5′-GGG ACT TTG CCA TAA AGA C-3′, Subtype B-1MM-2T: 5′-GGG ACT TTG CCA TAA AGA T-3′, and Subtype B-1MM-2A: 5′-GGG ACT TTG CCA TAA AGA A-3′. The subtype B-derived template used was: Subtype B-K65-ssDNA: 5′-AGT ACT GTC TTT T**(T)T CTT TAT GGC AAA GTC CCC** CCT TTT CTT TTA A-3′, where the bolding indicates primer binding regions.

The subtype C-derived primers used were: Subtype C-0MM-1: 5′-GGG GAC TTT GCT ATA AAA A-3′, Subtype C-1MM-2: 5′-GGG ACT TTG CTA TAA AAA G-3′, Subtype C-1MM-2C: 5′-GGG ACT TTG CTA TAA AAA C-3′, Subtype C-1MM-2T: 5′-GGG ACT TTG CTA TAA AAA T-3′, and Subtype C-1MM-2A: 5′-GGG ACT TTG CTA TAA AAA A-3′. The subtype C-derived template used was: Subtype C-K65-ssDNA: 5′-AGT ACT GTC TTT C**(T)T TTT TAT AGC AAA GTC CCC** CCT TTT CTT TTA A-3′. The bold nt on the template denote primer-binding regions. The underlined nt on the primers denote the bases responsible for incorrect base pairing. Nt in parentheses represent potential sites of K65R development and dislocation. It is important to note that the terminal “A” of the Subtype C-1MM-2A primer was not underlined because it is responsible for the production of wild-type, K65K-containing products as opposed to products with mutagenic nt insertions. A summary of all primers and templates used is provided in Table 2.

**Table 2:** Sequences of primers and templates derived from subtype B and C HIV-1 used to evaluate dislocation during DNA synthesis.

Subtype B Sequences	
B-0MM-1	5′-GGGGACTTTGCCATAAAGA-3′
B-1MM-2	5′-GGGACTTTGCCATAAAGAG-3′
B-1MM-2C	5′-GGGACTTTGCCATAAAGAC-3′
B-1MM-2T	5′-GGGACTTTGCCATAAAGAT-3′
B-1MM-2A	5′-GGGACTTTGCCATAAAGAA-3′
Subtype B-K65-ssDNA	5′-AGTACTGTCTTT**TTTCTTTATGGCAAAGTCCCC**CCTTTTCTTTTAA-3′

Subtype C Sequences	
C-0MM-1	5′-GGGGACTTTGCTATAAAAA-3′
C-1MM-2	5′-GGGACTTTGCTATAAAAAG-3′
C-1MM-2C	5′-GGGACTTTGCTATAAAAAC-3′
C-1MM-2T	5′-GGGACTTTGCTATAAAAAT-3′
C-1MM-2A	5′-GGGACTTTGCTATAAAAAA-3′
Subtype C-K65-ssDNA	5′-AGTACTGTCTTT**CTTTTTTATAGCAAAGTCCCC**CCTTTTCTTTTAA-3′

Bold sequences denote the primer binding nt span on the template. Underlined nt denote bases responsible for incorrect base pairing. ss, single-stranded.

All templates and primers were purified by electrophoresis on 8% polyacrylamide 7 M urea gels containing 50 mM Tris-borate pH 8 and 1 mM EDTA. The DNA templates were then eluted from gel slices using a buffer containing 500 mM NH_4_Ac and 0.1% SDS. 5′-end labeling of the primers was conducted with [γ^32^P]-ATP and T4 polynucleotide kinase according to the manufacturer's recommendations (Applied Biosystems/Ambion, Austin, TX, USA). Sequence alignments were performed using the BioEdit Biological Sequence Alignment Editor (Ibis Therapeutics, Calesbad, CA, USA).

### Expression and purification of recombinant subtype B and C RT

The enzymes used were heterodimeric recombinant subtype B and C (p66/p51) wild-type RTs that were expressed in *Escherichia coli* cells and purified as previously described [46], [59]. For all of the assays presented in the manuscript, we used single purified batches of subtype B RT and of subtype C RT to provide consistency in our data when comparing the subtype B vs. subtype C template reaction products.

### Primer-dependent and independent K65R production and template dislocation experiments

To study the generation of K65R in a primer-dependent manner, the primers used contained a mutagenic G at their 3′ end in order to yield K65R-containing transcripts. For subtypes B and C, the Subtype B-1MM-2 primer and Subtype C-1MM-2 primers, respectively, were used to give rise to K65R-containing transcripts. Briefly, 100 nM of the 5′-labeled primer were heat-annealed with 300 nM of template at 95°C for 2 min, 72°C for 20 min and at 37°C for 20 min. Once annealed, the DNA/DNA hybrids were incubated with 600 nM of HIV-1 RT in a buffer containing 50 mM Tris-HCl, pH 7.8, 50 mM NaCl, 6 mM MgCl_2_ and 10 µM each of the four dNTPs (Invitrogen, Carlsbad, CA, USA). The reaction was allowed to proceed at 37°C and was stopped by the addition of 20 µl of the reaction mix into 100 µl of 100% isopropyl alcohol containing 300 mM ammonium acetate and 10 ng/µl bulk Yeast RNA (Applied Biosystems/Ambion, Austin, TX, USA) at 0, 15, 30, 45, 60, 75, 90, 105 and 120 min. After the reaction, the DNA was resuspended in 10 µl of 100% formamide containing bromphenol blue and xylene cyanol. The samples were analyzed on 8% polyacrylamide 7 M urea gels containing 50 mM Tris-borate pH 8 and 1 mM EDTA. The results were analyzed by exposing the radioactive material to phosphor screens and phosphorimaging.

To study the template-derived generation of K65R in a primer-independent manner, we used primers that did not contain mutagenic nt but instead used only dGTP to exclusively yield transcripts containing the mutagenic nt. These reactions were carried out under conditions similar to those described above with the exception of the addition of 10 µM of dGTP only (Invitrogen, Carlsbad, CA, USA) as opposed to all four nt in the primer-dependent K65R generation assays.

Graphical representation and statistical analysis of results using a two-tailed Student's *t* test were performed with Prism 5 (GraphPad Software, La Jolla, CA, USA). To ensure the template-specific and enzyme-independent nature of the findings, the experiments were repeated a minimum of three times using both subtype-matched and subtype-mismatched wild-type RT enzymes.

### Primer-independent and dependent generation of K65R, K65T and K65I and template dislocation

To evaluate whether the erroneous incorporation of the other nt alternatives would influence dGTP incorporation and K65R production, we also performed the primer-independent DNA synthesis experiments in the presence of dCTP and dTTP, to yield K65T- and K65I-containing transcripts, respectively.

To ensure that the dislocation reaction was specific to the generation of the K65R mutation, primers were used that contained each of the three other possible nt at the mutagenic position at the 3′ end, such that mismatching with the template sequence would occur to yield K65K, K65T or K65I-containing transcripts in a primer-dependent manner.

Experiments were performed as described above with the addition of 10 µM of each of the dGTP, dCTP and dTTP nt (Invitrogen, Carlsbad, CA, USA) for the primer-independent reactions versus the addition of 10 µM of each of the four dNTPs (Invitrogen, Carlsbad, CA, USA) for the primer-dependent reactions.

### *In silico* RNA secondary structure prediction and analysis

RNA secondary structures were predicted and analyzed according to their Gibbs free energy properties using the Mfold RNA secondary structure program (http://mfold.burnet.edu.au/rna_form) [60]. The full-length RT sequences of NL4-3 subtype B HIV-1, MJ4 subtype C HIV-1 and SIVcpz were obtained from the Los Alamos National Laboratory HIV Sequence Database (http://www.hiv.lanl.gov/content/index) and several fragments lengths of each were analyzed. It is important to note that we were limited in our ability to compare sequences no larger than the size of the RT segment of *pol* with the *in silico* method that we employed. The linear RNA predictions with the three lowest Gibbs free energy predictions were compared at a folding temperature of 37°C and under divalent ion-free conditions in 1M NaCl. The maximum number of folds was limited to 50.

## Results

### Subtype B and C RT Sequence Analysis and Generation of K65R

To better understand the role of specific nt polymorphisms in the generation of drug resistance, we aligned and analyzed the entire nt and amino acid sequences of RT including codon K65 for both subtypes B and C. The subtype B and C sequences are approximately 90% and 93% homologous at the nt level (Figure S1A) and amino acid level (Figure S1B), respectively. Because a proportion of nt polymorphisms that distinguish the two subtypes do not result in changes of the amino acid sequence, it is not surprising that RTs derived from either subtype B or C are similar enzymatically [46], [47], [48], [50].

### Primer-dependent generation of K65R and template dislocation

**Figure 2:**
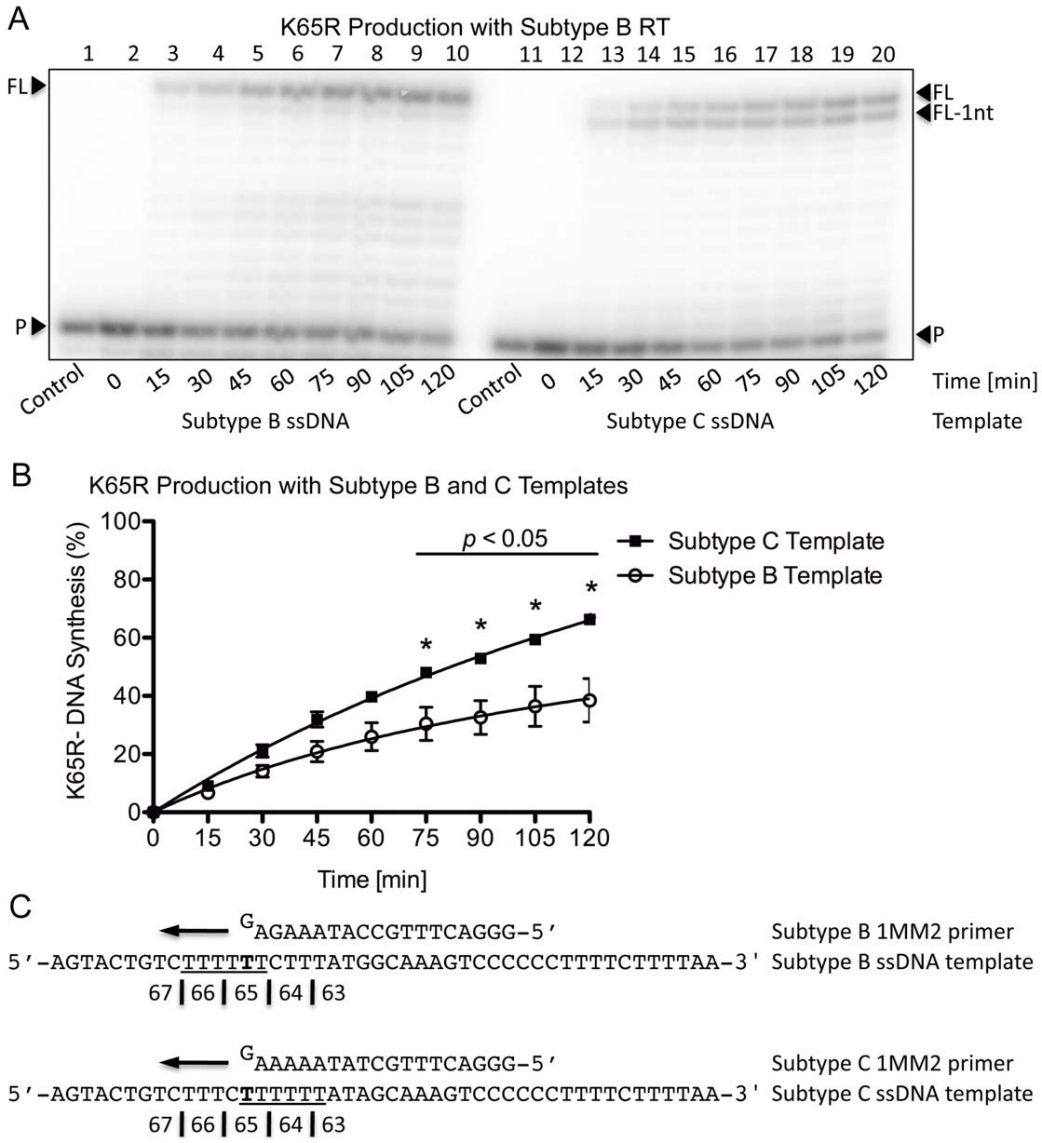
K65R production rates with subtype B RT on subtype B and C templates with primers containing the mutagenic nt. (A) Lanes 1 through 10 depict (+)dsDNA synthesis from the (-)ssDNA intermediate with subtype B RT on the subtype B template. The full-length product is observed as a single band at the FL position. Lanes 11 through 20 depict (+)dsDNA synthesis from the (−)ssDNA intermediate with subtype B RT on the subtype C template. The full-length product is observed as two distinct bands at the FL and FL-1nt positions, which is indicative of dislocation on the subtype C template. (B) Graphical representation of the amount of transcripts containing the mutagenic nt produced with subtype B RT on both subtype B and C templates. The values indicated with an asterisk have a *p*-value <0.05 when the amount of K65R-production between both subtypes at the given time-point is compared. More transcripts with the mutagenic G at position 65 are produced with the subtype C template than with the subtype B template. (C) Depiction of the primer and template systems used. The primers contain a G base on their 3′-end that becomes mismatched on the T on the template strand thus yielding transcripts with the mutagenic nt. The homopolymeric regions of both templates are underlined and the base responsible for the K65R mutation is indicated in bold.

The production of DNA transcripts that contained the mutagenic G at the central position of codon 65 was evaluated using the subtype B-1MM2 and C-1MM2 primers on their respective subtype-matched (−)ssDNA templates. (+)dsDNA synthesis was evaluated simultaneously on both subtype B and C templates using wild-type subtype B (BWT) RT (Figure 2A). With the subtype B template, no pausing was seen and a distinct, 13 nt DNA product band containing K65R was observed at the full-length (FL) position. In contrast, use of the subtype C template led to two distinct DNA product bands at the FL and full-length -1 nt (FL-1nt) positions of 13 and 12 nt respectively, suggesting that a probable primer/template misalignment had occurred. Quantification results showed that larger amounts of DNA transcripts containing the mutagenic G nt were produced from the subtype C than subtype B template (68% vs. 40%, *p*<0.05) after 120 min (Figure 2B). The subtype B and C 1MM2 primers used contained a G at their 3′-end that should mismatch opposite a T (Figure 2C). Since the nt immediately downstream of the T on the subtype C template is a C, the template probably folded onto itself at that position to allow for the 3′-G of the primer to bind to the C of the template strand. Thus, misalignment would result in dislocation to produce either transcripts with a -1 nt frameshift mutation (FL-1nt band) or, upon subsequent primer/template realignment, transcripts containing the K65R mutation (FL band). It is important to note that the full-length products (FL) in the context of the subtype C primer/template sequences could have also been the result of either a realigned dislocation or of direct mispair extension. However, the later scenario would be less likely to occur as is observed on the subtype B sequence where dislocation is not present and in the assessment of the pre- and post-realignment products presented later.

**Figure 3:**
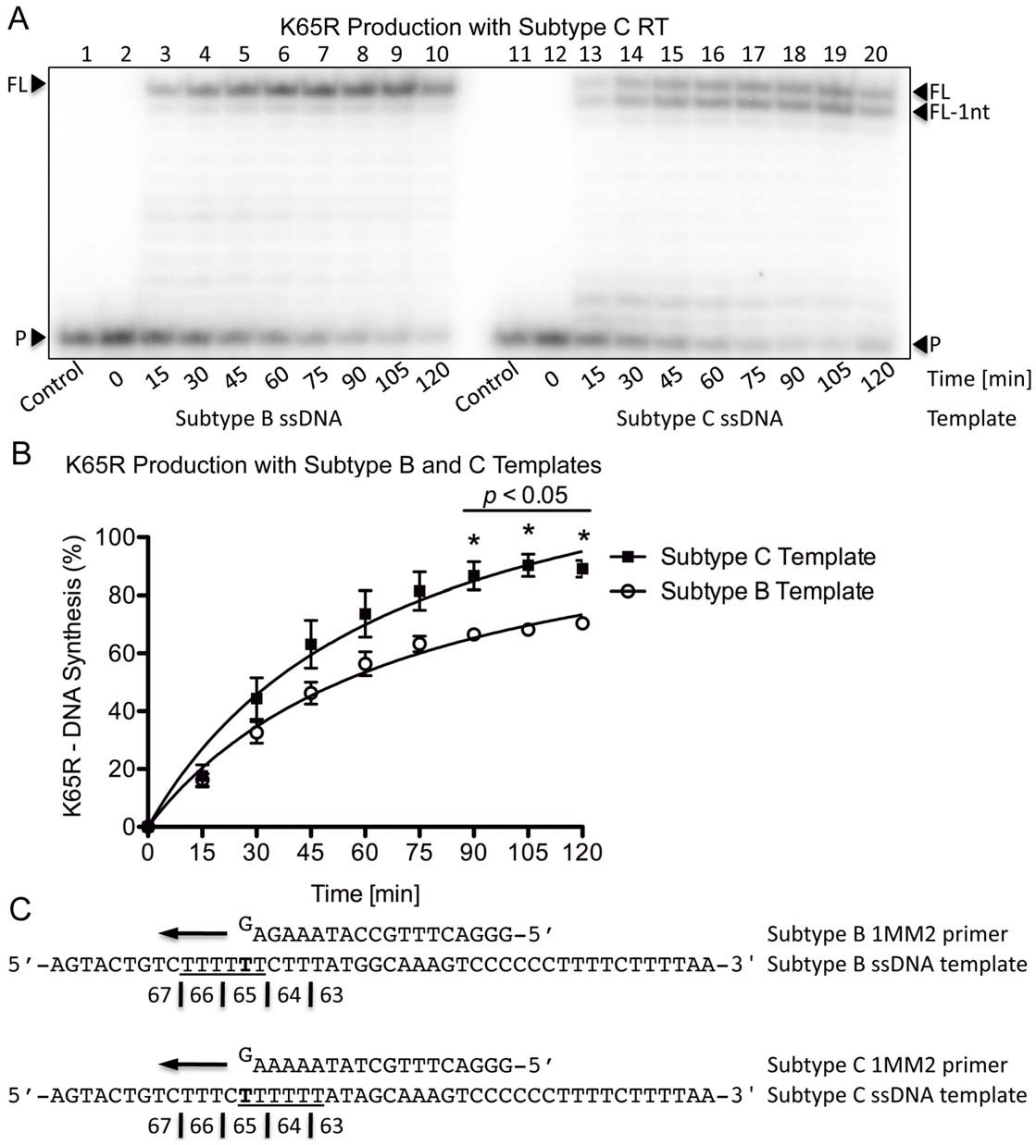
K65R production rates with subtype C RT on subtype B and C templates with primers containing the mutagenic nt. (A) Lanes 1 through 10 depict (+)dsDNA synthesis from the (−)ssDNA intermediate with subtype C RT on the subtype B template. The full-length product is observed as a single band at the FL position. Lanes 11 through 20 depict (+)dsDNA synthesis from the (−)ssDNA intermediate with subtype C RT on the subtype C template. The full-length product is observed as two distinct bands at the FL and FL-1nt positions, which is indicative of dislocation on the subtype C template. The results are similar to those obtained when subtype B RT was used on the same templates, thus highlighting the template-specific and enzyme-independent nature of the mechanism. (B) Graphical representation of the amount of transcript production with subtype C RT on both subtype B and C templates. The values indicated with an asterisk have a *p*-value <0.05 when the amount of K65R-production between both subtypes at the given time-point is compared. More transcripts containing the mutagenic nt are produced with the subtype C template than with the subtype B template. The same trend is present when subtype B RT was used on the same templates. (C) Depiction of the primer and template systems used. The primers contain a G base at their 3′-end that becomes mismatched on the T on the template strand thus yielding K65R-containing transcripts. The homopolymeric regions of both templates are underlined and the base responsible for the K65R mutation is indicated in bold.

To ensure the template-specific and enzyme-independent nature of both K65R production and primer/template dislocation on the subtype C template, similar experiments were performed using wild-type subtype C (CWT) RT. Similar patterns of DNA synthesis were observed as for the BWT RT reaction (Figure 3A) with the subtype C template again yielding more transcripts with the mutagenic nt than the subtype B template (95% vs. 70%, *p*<0.05) after 120 min (Figure 3B). A comparison of primer and template sequences shows that similar mechanisms of primer/template misalignment, slippage and dislocation are the likely cause of the elevated rates of K65R with the subtype C template (Figure 3C).

In contradistinction to the major differences obtained between the use of templates of subtype B or C origin in formation of K65R transcripts, the relatively minor differences obtained with subtype B vs. subtype C RTs are probably the result of the different purification activities of the enzymes used.

### Primer-independent K65R production and template dislocation

**Figure 4:**
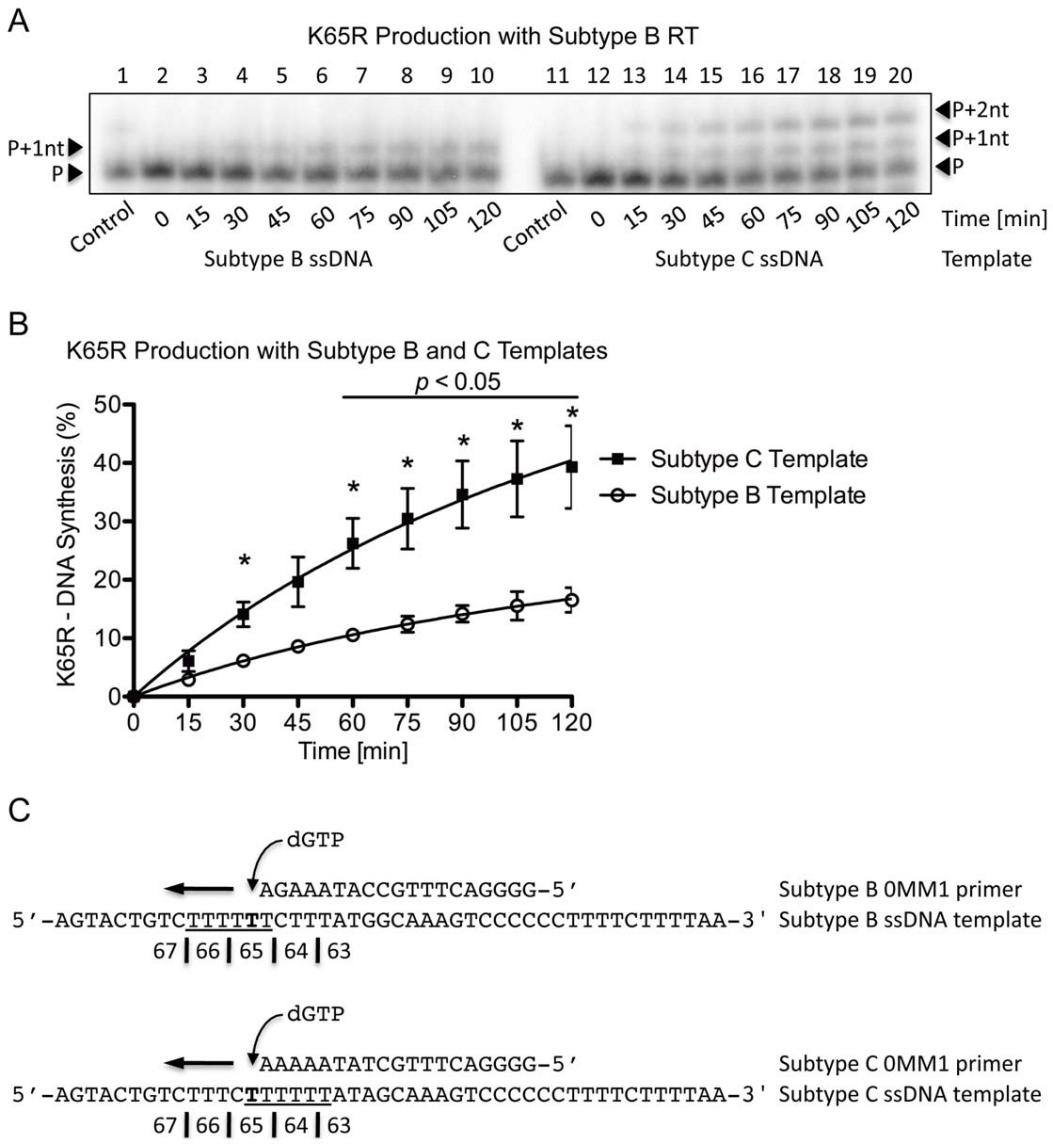
Single incorrect nt incorporation and K65R production rates with subtype B RT on subtype B and C templates. (A) Lanes 1 through 10 depict (+)dsDNA synthesis from the (−)ssDNA intermediate with subtype B RT on the subtype B template. The full-length product is observed as a single dGTP incorporation occurred at the P+1nt position. Lanes 11 through 20 depict (+)dsDNA synthesis from the (−)ssDNA intermediate with subtype B RT on the subtype C template. The full-length product is observed as containing two distinct dGTP incorporations at the P+1nt and P+2nt positions, indicative of dislocation with the subtype C template. (B) Graphical representation of transcript production with subtype B RT on both the subtype B and C templates. The values indicated with an asterisk have a *p*-value <0.05 when the amount of transcripts containing the mutagenic nt produced between both subtypes at the given time-point is compared. More transcripts containing the mutagenic G nt at codon 65 are produced with the subtype C template than with the subtype B template. (C) Depiction of the primer and template systems used. dGTP was the only nt employed in the reaction to allow for the production of the mutagenic transcripts. The homopolymeric regions of both templates are underlined and the base responsible for the K65R mutation is indicated in bold.

To evaluate the production of DNA transcripts containing the mutagenic nt and to identify the exact location of dislocation on the subtype C template, (+)dsDNA synthesis was evaluated using the Subtype B-0MM1 and C-0MM1 primers on their respective, subtype-matched (−)ssDNA templates. As stated, the 0MM1 primers did not contain a mutagenic nt at the 3′ position and dGTP was the only nt provided to ensure production of K65R-containing transcripts. Only a single band was present at the primer +1 nt (P+1nt) for the subtype B template sequence whereas a double band was present at the P+1nt and P+2nt positions with the subtype C template (Figure 4A). This is indicative of primer/template slippage misalignment and confirms that the dislocation occurred at the K65 site. As anticipated, more transcripts containing the mutagenic G nt were produced from the subtype C than subtype B template (40% vs. 15%, *p*<0.05) after 120 min (Figure 4B). Although fewer DNA transcripts containing the mutagenic nt were present than in the primer-dependent reaction, this may reflect additional thermodynamic energy barriers necessary for the primer-independent reaction that requires misincorporation of a dGTP opposite a T on the template. When comparing the subtype B and C primers and templates in the primer-independent setting, dislocation occurred only with the subtype C template at the exact location of K65R-development (Figure 4C), probably because the dislocation allowed the primer and template to misalign, permitting dGTP to correctly bind opposite the C to yield the P+1nt product. The P+2nt product was obtained when the primer and template realigned and allowed for a second dGTP to be incorporated immediately following the first G on the subtype C but not B template. As described earlier, direct misincorporation, although less likely considering the template-specific conditions, could have also produced the band at position P+2nt. Indeed, the differences observed between the subtype B and C templates with regard to the development of transcripts containing the mutagenic nt are likely due to the added propensity for dislocation mutagenesis to occur on the subtype C template.

**Figure 5:**
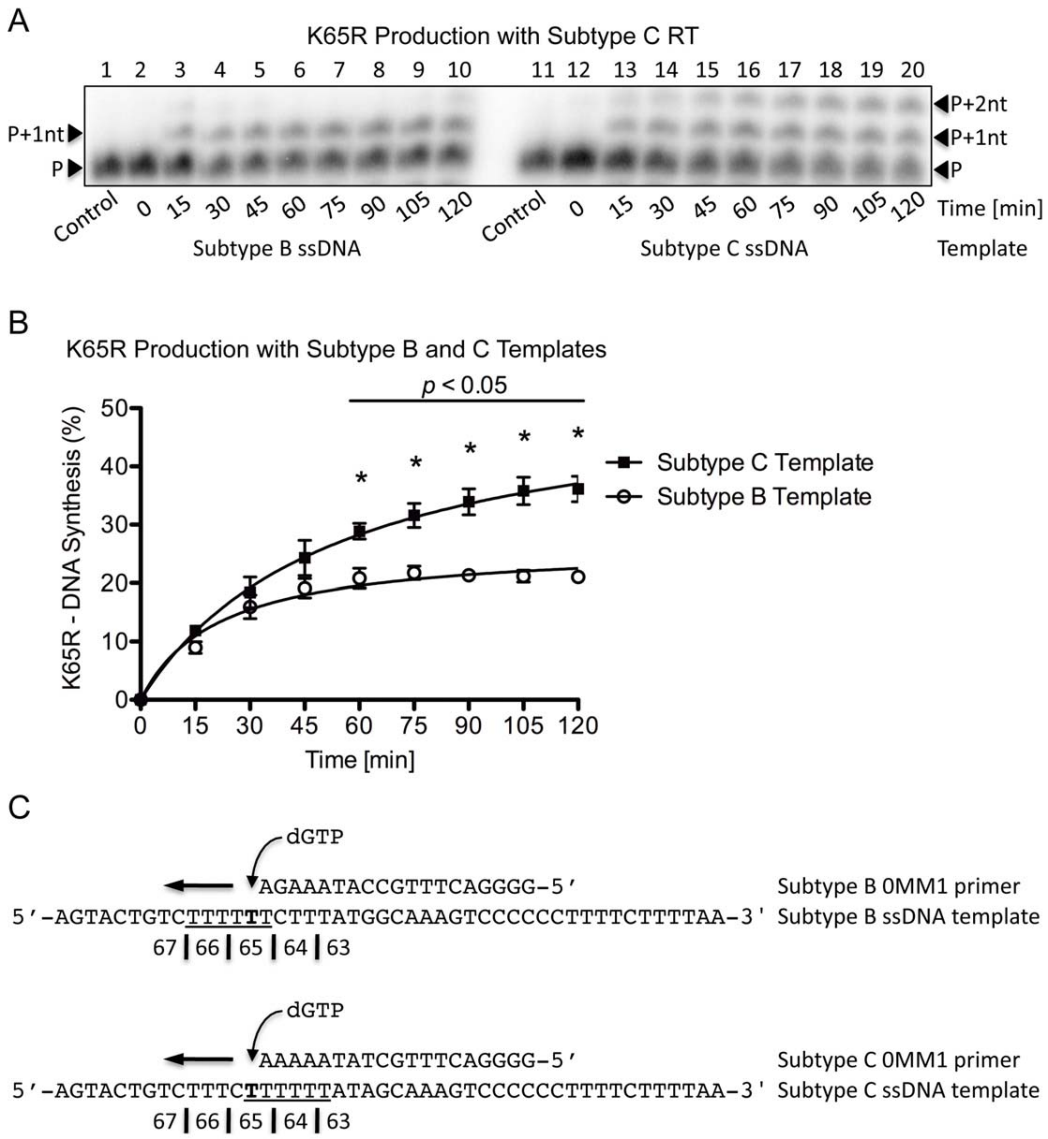
Single incorrect nt incorporation and K65R production rates with subtype C RT on subtype B and C templates. (A) Lanes 1 through 10 depict (+)dsDNA synthesis from the (−)ssDNA intermediate with subtype C RT on the subtype B template. The full-length product contains a single dGTP incorporation at the P+1nt position. Lanes 11 through 20 depict (+)dsDNA synthesis from the (−)ssDNA intermediate with subtype C RT on the subtype C template. The full-length product contains two distinct dGTP incorporations at the P+1nt and P+2nt positions, indicative of dislocation with the subtype C template. (B) Graphical representation of transcript production with subtype C RT on both the subtype B and C templates. The same trend as when the subtype B-derived RT enzyme was employed was observed, further reinforcing the template-specific and enzyme independent notion of the mechanism. (C) Depiction of the primer and template systems used. The homopolymeric regions of both templates are underlined and the base responsible for the K65R mutation is indicated in bold.

To reaffirm that the findings were specific to the subtype origin of the template and not the RT, the same reactions were performed using CWT RT (Figure 5). Similar patterns of DNA synthesis were seen as with CWT RT on both the subtype B and C templates (Figure 5A) and more transcripts were again produced on the subtype C template after 120 min (37% vs. 22%, *p*<0.05) (Figure 5B). Again, dislocation occurred at the K65 site only when the subtype C template and primer were employed (Figure 5C).

As with the primer-dependent assays, the different purification activities of the enzymes used are likely contributors to the relatively minor differences in K65R-transcript production when subtype B RT is directly compared to subtype C RT. Again, this is in contrast to the major differences that result from templates of either subtype B or C origin.

### Template dislocation and specificity of dGTP incorporation

**Figure 6:**
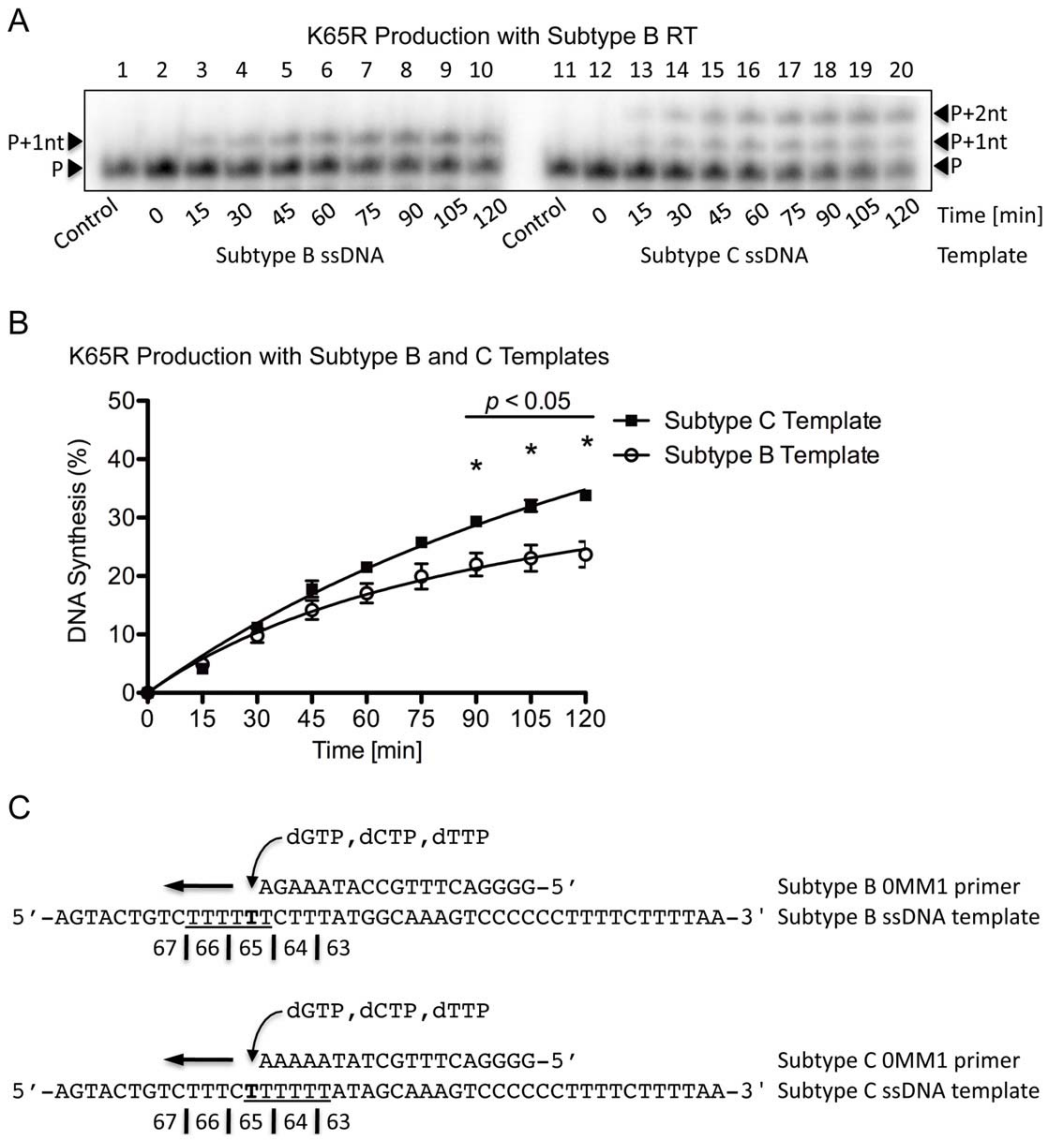
Multiple incorrect nt incorporations at the K65 position with subtype B RT on subtype B and C templates. (A) Lanes 1 through 10 depict (+)dsDNA synthesis from the (−)ssDNA intermediate with subtype B RT on the subtype B template. The full-length product contains a single dGTP, dCTP or dTTP incorporation opposite the T in the template strand at the P+1nt position. Lanes 11 through 20 depict (+)dsDNA synthesis from the (−)ssDNA intermediate with subtype B RT on the subtype C template. Dislocation is observed with the subtype C template as two distinct nt incorporations at the P+1nt and P+2nt positions. (B) Graphical representation of DNA synthesis with subtype B RT on the subtype B and C templates. The values indicated with an asterisk have a *p*-value <0.05 when the amount of transcript-production between both subtypes at the given time-point is compared. (C) Depiction of the primer, template and nt employed in the system. The homopolymeric regions of both templates are underlined and the base responsible for the K65R generation is bolded.

To evaluate the mutagenic potential of the two other nt that might be incorrectly incorporated at the K65 site, we conducted RT reactions in the presence of dCTP, dTTP and dGTP. When BWT RT was employed with either the subtype B and C primer/template systems, the characteristic double-banding pattern of dislocation was only seen with the subtype C template (Figure 6A). More transcripts were again produced with the subtype C than subtype B template after 120 min (35% vs. 22%, *p*<0.05) (Figure 6B). In the presence of dCTP, dTTP or dGTP, it is only dGTP incorporation on a dislocated subtype C primer/template that might simultaneously produce a band at the P+1nt position and, upon realignment, a band at the P+2nt position, which would have allowed a second dGTP to be correctly incorporated (Figure 6C). We speculated that addition of dCTP and dTTP to the reaction would cause the P+1nt band produced from the C template to diminish significantly if dislocation at this position was not specific to dGTP. In this context, dCTP or dTTP could occupy the P+1nt position and allow for correct dGTP binding to occur at the P+2nt position, thereby eliminating the majority of P+1nt product. However, this did not occur, showing that dGTP is preferentially incorporated over other nt at the K65 site on the subtype C template via primer/template slippage and dislocation mutagenesis.

**Figure 7:**
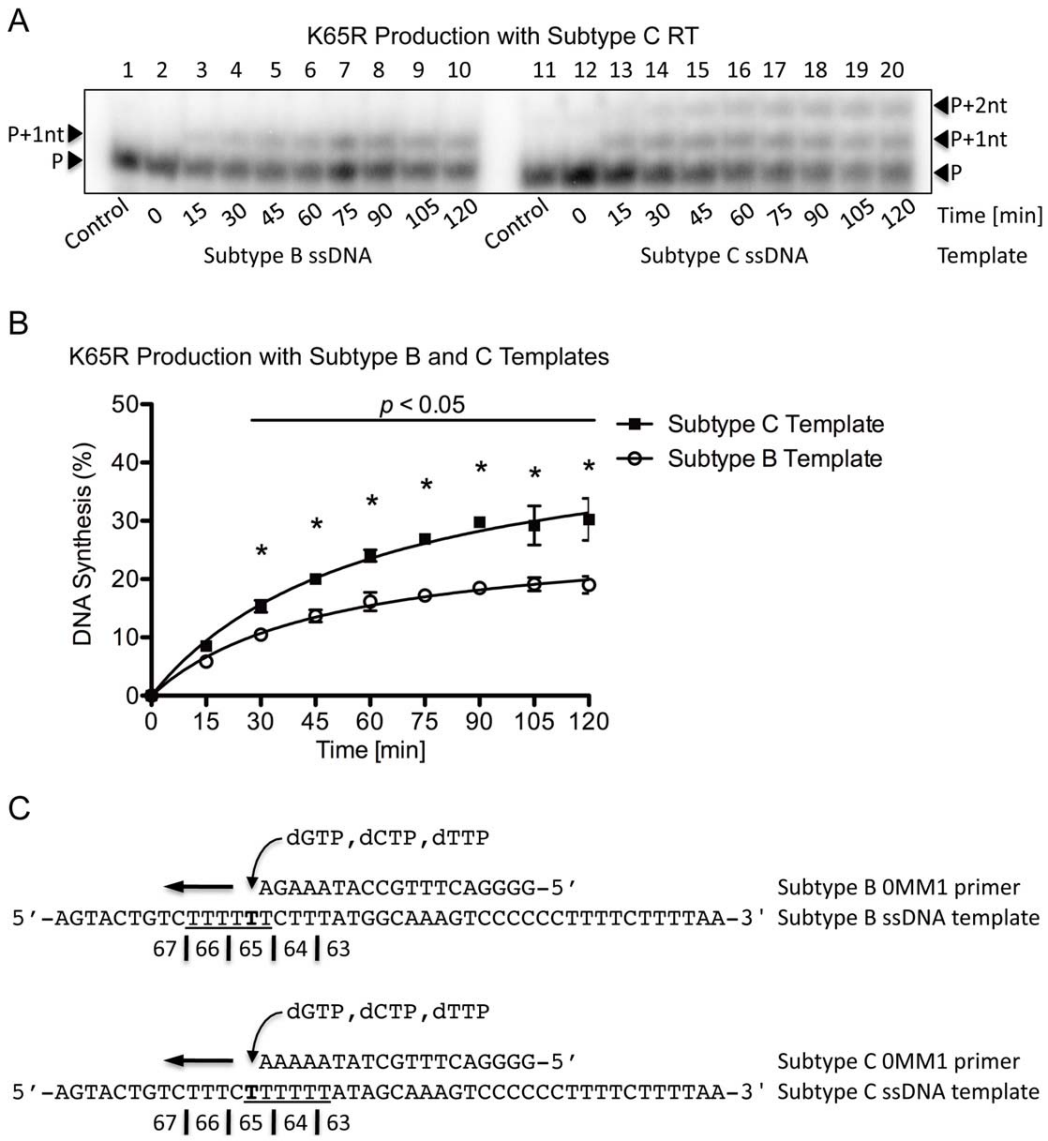
Multiple incorrect nt incorporations at the K65 position with subtype C RT on subtype B and C templates. (A) Lanes 1 through 10 depict (+)dsDNA synthesis from the (−)ssDNA intermediate with subtype C RT on the subtype B template. The full-length product is observed as a single dGTP, dCTP or dTTP incorporation occurred opposite the T in the template strand at the P+1nt position. Lanes 11 through 20 depict (+)dsDNA synthesis from the (−)ssDNA intermediate with subtype C RT on the subtype C template. Dislocation is only present with the subtype C template. (B) Graphical representation of DNA synthesis with subtype C RT on the subtype B and C templates. The same trend is observed regardless of origin of the RT enzyme used. (C) Depiction of the primer, template and nt used in the reaction. The homopolymeric regions are underlined and the base responsible for the K65R mutation is indicated in bold.

Similar levels of P+1nt and P+2nt products were obtained when CWT RT was used for the reaction in the presence of dCTP, dTTP and dGTP (Figure 7A) and the subtype C templates produced more transcripts than the subtype B transcripts after 120 min (30% vs. 18%, *p*<0.05) (Figure 7B). Thus, dGTP is preferentially and erroneously incorporated at the K65 site despite the presence of other mutagenic nt (Figure 7C).

### Assessment of pre and post-realignment products following primer/template dislocation

**Figure 8:**
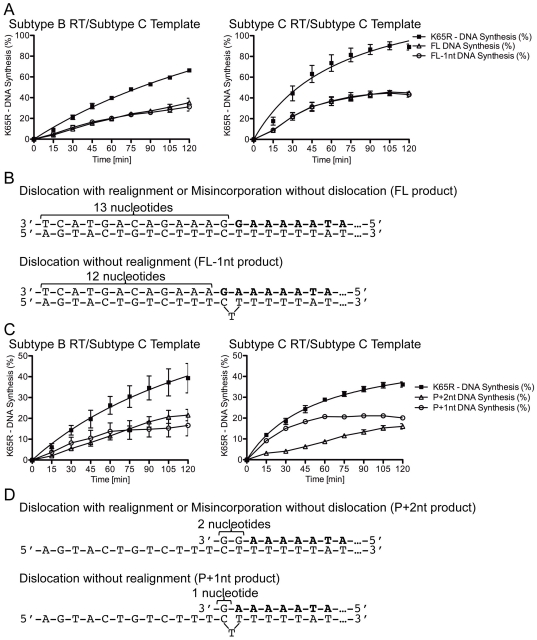
Evaluation of the dislocation products in the various subtype C primer/template conditions. (A) Graphical representation of K65R-containing transcript production with primers already harboring the mutagenic nt. *Left*: K65R production with subtype B RT on the subtype C template. *Right*: K65R production with subtype C RT on the subtype C template. The product of dislocation without realignment (FL-1nt product) is depicted as empty circles and the products of dislocation with realignment or misincorporation without dislocation (FL product) are depicted as empty triangles. When the mutagenic nt is already incorporated into the primer strand, the amount of FL and FL-1nt products is identical. (B) Schematic of the dislocation without realignment (FL-1nt) and the dislocation with realignment or misincorporation without dislocation (FL) products with the primer strand already containing the mutagenic nt. The sequence of the unextended primer is depicted in bold. (C) Graphical representation of K65R-containing transcript production with dGTP incorporation at the site of K65R development. *Left*: K65R production with subtype B RT on the subtype C template. *Right*: K65R production with subtype C RT on the subtype C template. The product of dislocation without realignment (P+1nt product) is depicted as empty circles and the products of dislocation with realignment or misincorporation without dislocation (P+2nt product) are depicted as empty triangles. During the process of incorporation of the mutagenic nt, the amount of P+1nt product reaches a maximum at 60 min whereas the amount of P+2nt product continues to rise steadily beyond 60 min. (D) Schematic of the dislocation without realignment (P+1nt) and the dislocation with realignment or misincorporation without dislocation (P+2nt) products during dGTP incorporation at the site of K65R development. The sequence of the unextended primer is depicted in bold.

The levels of pre and post-realignment products following dislocation can shed light on the proportion of transcripts that contain the K65R mutation or that harbor a -1 frameshift mutation during primer-dependent transcription from the subtype C template. The data of Figure 8A show that the amount of transcripts that resulted from the primer and template that have not realigned (FL-1nt products) is equivalent to that which has realigned (FL products). With BWT RT, 36% of transcripts contained the FL product and 35% contained the FL-1nt product (Figure 8A, left panel). In the case of CWT RT, the FL and FL-1 products each represent 41% of the transcripts (Figure 8A, right panel). Dislocation and realignment will yield a 13 nt product that corresponds to the band at the FL position (Figure 8B). All of the transcripts that resulted from dislocation and subsequent realignment contained K65R. As discussed earlier, misincorporation of the 3′-G of the primer sequence opposite the T in the absence of dislocation might also generate, although with lower probability, the 13 nt FL product. In contrast, dislocation that is not followed by realignment will yield a 12 nt product that corresponds to the band at the FL-1 position (Figure 8B). These transcripts contain a -1 frameshift mutation with a deletion of the A at the middle position of codon 65.

In the primer-independent experiments, where dGTP was the only nt provided to evaluate dislocation, different results were obtained. When using BWT RT or CWT RT on the subtype C template, the levels of transcripts resulting from primers and templates that had not realigned (P+1nt products) steadily increased over 60 min, after which these levels remained constant despite increases in the total amount of the P+2nt realignment product and total DNA product (Figure 8C). This suggests that non-realigned products (P+1nt products) might eventually realign to produce more K65R-containing transcripts (P+2nt products). In the case of the BWT RT reaction, the non-realigned (P+1nt) products plateaued at 18% (Figure 8C, left panel) versus 21% for the CWT RT reaction (Figure 8C, right panel).

The realigned products (P+2nt) showed similar and constant increases throughout the duration of the reaction for both BWT and CWT RT. In the primer-independent reaction, dislocation that occurs with realignment yields a 2 nt product at the P+2nt position that contains intact K65R transcripts (Figure 8D). When dislocation occurs and is not followed by realignment, a single nt product is formed at the P+1nt position (Figure 8D). These transcripts harbor a -1 frameshift mutation containing a deletion of the A at the middle position of codon 65. Over time, production of P+1nt products seems to level off as a result of realignment to form additional P+2nt products that contain the K65R mutation.

Similar findings were observed with the addition of dCTP and dTTP to the primer-dependent system with regard to the production and maintenance of the P+1nt and P+2nt products (Figure S2A), and with regard to the realigned and non-realigned dislocation products (Figure S2B). The band at position P+1nt did not diminish with the addition of dCTP and dTTP in the system, and the amount of P+1nt product on the subtype C template was similar to that when only dGTP was used for the reaction (Figure S2A). These observations confirm and extend the notion that dGTP is preferentially incorporated at the K65 site due to the dislocation potential of the template at this position.

### Dislocation specificity and generation of K65K, K65T and K65I

**Figure 9:**
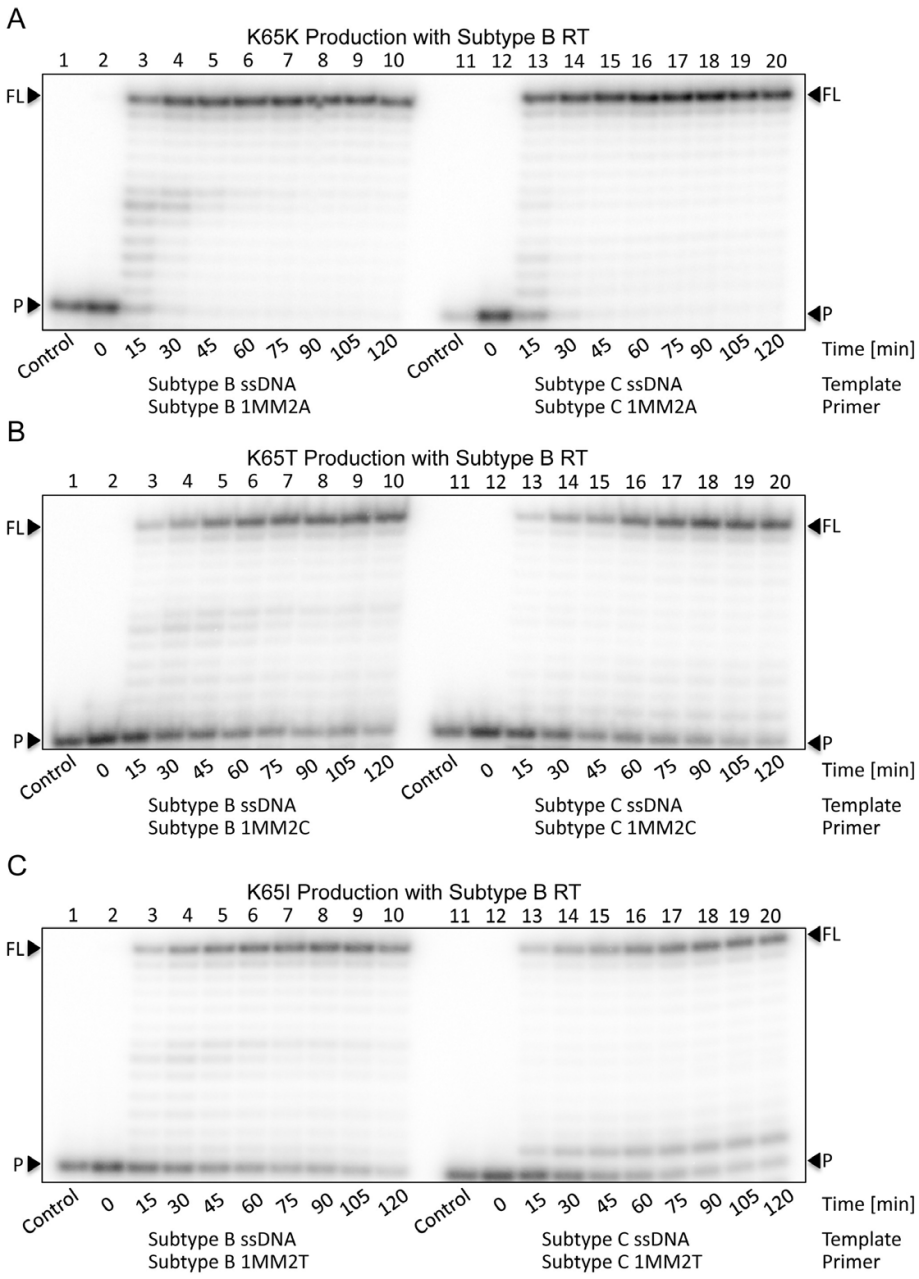
K65K, K65T and K65I production as a result of DNA synthesis with primers containing the three nt alternatives and their impact on dislocation. (A) Lanes 1 through 10 depict (+)dsDNA synthesis from the (−)ssDNA intermediate with subtype B RT on the subtype B template. The full-length product is observed as a single band at the FL position. Lanes 11 through 20 depict (+)dsDNA synthesis from the (−)ssDNA intermediate with subtype B RT on the subtype C template. The full-length product is observed again as a distinct band at the FL and positions without any dislocation products. When A is present at the 3′-end of the primer strand, it correctly binds with the T of the template strand to yield wild-type K65K-containing transcripts. (B) Lanes 1 through 10 depict (+)dsDNA synthesis from the (−)ssDNA intermediate with subtype B RT on the subtype B template. The full-length product is observed as a single band at the FL position. Lanes 11 through 20 depict (+)dsDNA synthesis from the (−)ssDNA intermediate with subtype B RT on the subtype C template. No dislocation products were observed. When C is present at the 3′-end of the primer strand, it incorrectly binds with the T of the template strand to yield K65T-containing transcripts. (C) Lanes 1 through 10 depict (+)dsDNA synthesis from the (−)ssDNA intermediate with subtype B RT on the subtype B template. The full-length product is observed as a single band at the FL position without dislocation. Lanes 11 through 20 depict (+)dsDNA synthesis from the (−)ssDNA intermediate with subtype B RT on the subtype C template. When T is present at the 3′-end of the primer strand, it incorrectly binds with the T of the template strand to yield K65I-containing transcripts. Dislocation products are only observed when G is included on the primer strand yielding K65R-containing transcripts.

To confirm that dislocation only occurs with dGTP, primers containing the three other bases at their 3′-end were used to determine whether dislocation would occur at the K65 site during DNA synthesis. We first evaluated wild-type K65K production by using an A base at the 3′-end of the primer with BWT RT on both the subtype B and C templates. The data show that the 13 nt FL product was present as a distinct single band when either the subtype B or C templates were used (Figure 9A). The absence of the double band in the context of the subtype C template indicates that dislocation does not occur at K65 when the primer contains an A at its 3′-end. Instead, complete primer extension was accomplished early (15 min) in the reaction because no incorrect incorporations or mismatches were necessary for the completion of DNA synthesis.

K65T production was next evaluated with the addition of a C at the 3′-end of the primer with BWT RT on both the subtype B and C templates (Figure 9B). Again, a 13 nt product was observed with both templates without any evidence of dislocation. However, DNA synthesis did not occur as quickly as with synthesis of K65K because a mismatch was required for DNA synthesis to proceed beyond the +1 position on the template. Similarly, production of K65I, resulting from the presence of a T at the 3′-end of the primer, was similar for both templates, without any evidence of dislocation (Figure 9C). The 13 nt product formed more slowly than was the case for K65K due to the nt mismatch between the primer and template strands. Importantly, K65R and K65N are the only known N(t)RTI resistance-associated mutations at codon 65 of RT. K65T and K65I are very rare and viruses harboring either of these mutations remain fully susceptible to N(t)RTIs and NNRTIs. Nearly identical patterns to the BWT RT-mediated DNA synthesis were obtained with CWT RT for each of K65K (Figure S3A), K65T (Figure S3B), and K65I (Figure S3C). Thus, dGTP is the only nt that is capable of dislocation mutagenesis at the K65 site.

### Prediction and analysis of RNA secondary structure

The RNA secondary structures of the K65 region of HIV-1 subtype B, HIV-1 subtype C and SIVcpz were predicted and evaluated using the Mfold RNA secondary structure program [60]. When the RNA secondary structure of the region surrounding codon 65 of subtype B RT was predicted (Figure S4A), it corresponded exactly to published HIV-1 RNA secondary structure [61], with K65 being located in the unpaired region of a stem-loop structure. When subtype C RT RNA secondary structure in the same region was predicted, the structures obtained that had the two lowest Gibbs free energy values were similar to those of subtype B RT (Figure S4B), with K65 being located either in the unpaired loop of a stem-loop structure or in an unpaired bulge (Table 3). Both the unpaired loop and unpaired bulge RNA structures have high mutagenic potential and are considered to be “hot spots” for occurrence of drug resistance [62]. The secondary structure of the SIVcpz RNA was also predicted and analyzed because codons 64, 65 and 66 of SIVcpz harbor the same polymorphisms found in subtype C HIV-1 (Table 1). In addition, SIVcpz shares only 78% nucleic acid homology and 88% amino acid homology with subtype B HIV-1. In contrast to both subtype B and C HIV-1, the K65 region of SIVcpz was predicted to be present either between an unpaired loop and paired stem structure or in a paired stem structure (Figure S4C). These findings suggest that the silent nt polymorphisms that exist in subtype C HIV-1 may not significantly alter RNA secondary structure and are in agreement with previous biochemical data that focused on RNA as opposed to DNA templates. The former did not show significant differences in regard to K65R mutagenesis [48], [49], [50].

**Table 3:** Subtype B and C HIV-1 prediction and analysis of RNA secondary structure.

RNA Sequence	NucleotideHomology* †	Amino AcidHomology* †	ΔG 1‡	ΔG 2‡	ΔΔG 1*	ΔΔG 2*	K65 location
Subtype BHIV-1	100%	100%	-65.7	-	0.0	0.0	In the unpaired loop of a stem-loop structure
Subtype CHIV-1	90%	93%	-64.6	-63.4	1.1	2.3	In the unpaired loop of a stem-loop structure or in an unpaired bulge
SIV cpz	78%	88%	-66.6	-65.7	0.9	0.0	Between an unpaired loop and a paired stem structure or in a paired stem structure

*Nt homology, amino acid homology and differences in Gibbs free energy values of subtype C are compared to those of subtype B.

‡Gibbs free energy values are expressed in kcal/mole.

†Nt and amino acid homology was evaluated only for the RT portion of the *pol* gene.

## Discussion

We have previously described that a homopolymeric nt stretch is present in both subtype B and C HIV-1 RT coding sequences and that RT exhibits characteristic pausing events at the end of the sequence [48], [50]. Strong termination events with HIV-1 RT at the end of homopolymeric sequences have been associated with both template-primer misalignment frameshift deletion errors and dislocation/realignment substitution errors [54], [63], [64], [65]. In subtype C, a strong pause site was observed at the exact nt position responsible for K65R development and may be the cause for facilitated K65R development in subtype C viruses [48], [50]. These findings were specific to the subtype C template sequence and were not influenced by the subtype origin of the RT enzyme employed. Introduction of two silent subtype C nt polymorphisms into a subtype B NL4-3 virus, such that the homopolymeric sequence ends at the K65 site, rendered the subtype B virus hypersusceptible to K65R development [49].

**Figure 10:**
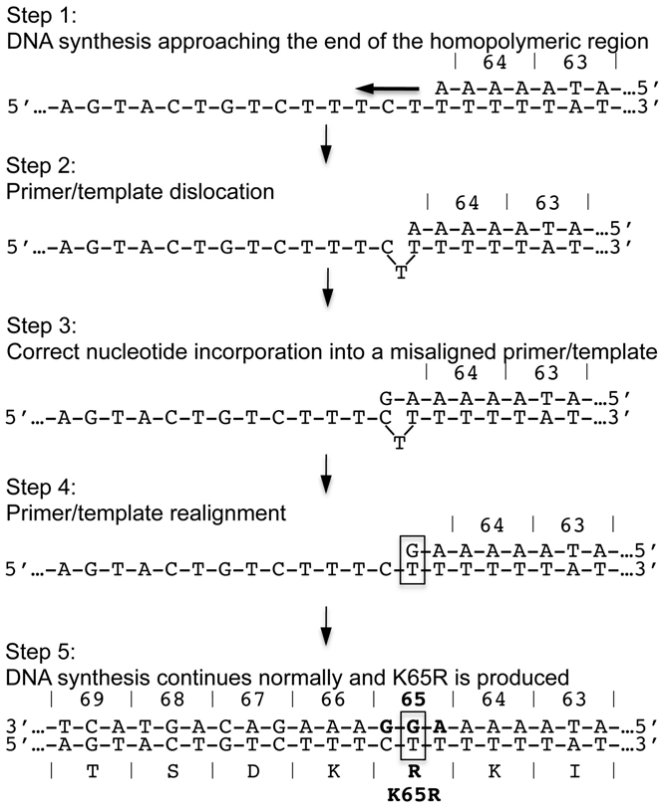
Schematic of dislocation mutagenesis and the development of the K65R mutation in subtype C HIV-1. *Step 1*: DNA synthesis approaches the end of a homopolymeric nt stretch that ends precisely at the location of K65R development. *Step 2*: At the end of the sequence, the RT enzyme exhibits characteristic pausing of DNA synthesis. The template-strand folds onto itself and exposes a C in the folded-over template strand. *Step 3*: A dGTP nt correctly binds opposite the C base of the misaligned template strand as DNA synthesis continues. *Step 4*: The primer and template strands realign and the same C base becomes re-exposed on the now correctly aligned template strand. *Step 5*: A second dGTP becomes incorporated opposite the re-exposed C on the correctly aligned primer/template strands and DNA synthesis continues normally. This series of events yields the AAG-to-AGG change that is responsible for the more facilitated appearance of the K65R mutation in subtype C HIV-1.

We have now explored the mechanisms responsible for these observations as described in Figure 10. Initially, RT synthesizes (+)dsDNA from a subtype C (−)ssDNA intermediate template. As the enzyme approaches the K65 site, it encounters a homopolymeric stretch of 6 T nt followed by a single C immediately adjacent to the site of K65R development. The two nt polymorphisms that are present in subtype C but not B are responsible for this positioning of the homopolymeric stretch in subtype C HIV-1, resulting in pausing and slippage of the primer and template strands. During slippage, the template folds over itself, resulting in misalignment of the template and primer, concealing the final T of the homopolymeric stretch and prematurely uncovering the following C. Hydrogen bonding provided by the incoming nt might then enhance the stability of the misaligned primer and template, resulting in a G being correctly introduced into the newly synthesized primer strand opposite the exposed C on the misaligned template. These events will produce DNA transcripts containing the -1 frameshift mutation that involves deletion of the middle A of codon K65. These findings are consistent with the one-base frameshift mutations that result from a well-described and characterized Streisinger strand slippage mechanism [66], [67].

Two alternative scenarios may then ensue. First, DNA synthesis may resume normally on the misaligned template and primer, yielding products containing the -1 frameshift. Alternatively, the primer and template strands might realign, following the incorporation of dGTP opposite the exposed C on the folded template, such that this C becomes available to bind to another incoming nt. As a result, a second dGTP nt might become incorporated into the primer strand immediately adjacent to the first dGTP and be mismatched opposite the T on the template. The single-base substitution at the end of a homopolymeric sequence following a Streisinger strand slippage is possible via dislocation mutagenesis that RT is known to display when encountering such sequences [54], [63], [64], [65], [68]. The single-base substitution in the context of subtype C HIV-1, following dislocation of the primer and template, is responsible for the AAG-to-AGG substitution, giving rise to the K65R drug resistance mutation.

With subtype C templates, reaction products included either a -1 frameshift mutation that deleted the middle A at codon K65 or full-length K65R-containing products. These products were characterized by distinct double banding patterns indicative of dislocation mutagenesis for both primer-dependent and primer-independent reactions. There is also the possibility of a -5 nt frameshift, deleting the same middle A of codon 65 in addition to the following G and the three following As prior to reaching the next template C. Dislocation was only observed when dGTP was incorporated into the misaligned template and primer as either a free nt or one already bound to the primer strand. Incorrect incorporation of dCTP or dTTP at the same site did not produce dislocation, indicating the importance of the nt flanking the template homopolymeric sequence. Had there been an A instead of a C flanking the homopolymeric template sequence, dislocation would not have occurred with dGTP but rather with dTTP instead. Thus, the length and base composition of the homopolymeric sequence as well as the bases that immediately follow it are all important in this process.

In addition, our data suggest that approximately half of the products obtained from the subtype C reactions contain full-length K65R-containing transcripts whereas the other half contain the -1 frameshft mutation. *In vivo*, viruses containing the -1 frameshift mutation are not viable and are unlikely to be detected in patients. As a result, the contribution of K65R-containing viruses that have resulted from the realigned dislocation reaction and that contain in-frame transcripts become more important in the clinical setting.

All of the reactions in which we showed Streisinger strand slippage-mediated frameshift mutations and dislocation-mediated single-base substitutions were performed using WT RT enzymes and WT subtype B and C HIV-1 templates. Newly infected people all harbor K65R minority species that result from spontaneous mutagenesis, although some may also have acquired this mutation by sexual transmission or by exposure to contaminated blood products [69], [70]. Such minority species are generally undetectable by standard genotyping due to overgrowth of WT viruses that have higher replication capacities than the mutated species in the absence of drug pressure.

Our findings are consistent with recent work on the role of the specific subtype C coding region surrounding codon 65 in the generation of K65R-containing viral minority species [58]. This work showed that the errors that result in K65R generation are not limited to HIV-1 RT but occur as well with other high-fidelity polymerase enzymes used in ultra-deep pyrosequencing reactions. However, the enzymes used in these assays had enhanced proofreading capabilities, thereby making them less likely to produce simple misinsertion errors when compared to HIV-1 RT. As a result, it is unlikely that K65R was generated as a result of simple misincorporation; instead, dislocation with realignment would have been the most probable mechanism whereby these enzymes might introduce base substitution mutations responsible for K65R development at the end of the homopolymeric sequence. These data, along with our evidence for sequence and subtype-specific dislocation, suggest that the HIV-1 subtype C virus has evolved to possess a “mutational hot-spot” at the site of K65R development that is not seen in subtype B. Strong termination by RT during DNA synthesis at this location, followed by dislocation-mediated base substitution, are the likely events that explain the rapid appearance of K65R in subtype C HIV-1.

Several recent reports have evaluated the presence of K65R in subtype C-infected individuals and have used appropriate measures to minimize the appearance of PCR-induced false-positive K65R-containing minority variants. In one of these studies, the authors showed by allele-specific PCR that K65R was present in 4 of 30 subtype C samples after treatment failure with N(t)RTIs but not in equivalent subtype B specimens [71]. Another study used high-fidelity DNA polymerase and sensitive real-time PCR assays in treatment-naïve individuals infected with subtype C HIV-1 and found that 6% of women and 15% of infants harbored K65R sequences as well as -1 frameshift mutants in which deletions of the A at the middle position of codon K65 were also found [72]. None of the infected infants had mothers whose viruses contained the K65R mutation, suggesting that the mutations had developed spontaneously. Two recent reports also suggest that low-level K65R containing variants are more frequent in subtype C- than B-infected patients [73], [74].

Subtype C-infected patients who harbor K65R minority species because of dislocation mutagenesis seem to be at higher risk for development of K65R drug resistance. These findings are particularly pertinent in subtype C endemic areas, including sub-Saharan Africa and India where most HIV-infected individuals are treated with the combination of lamividine, stavudine and nevirapine [37], [75]. A recent study evaluating the impact of minority species harboring NNRTI resistance mutations showed that patients harboring these viruses were more likely to fail NNRTI-containing ART regimens [76].

Another interesting consideration is whether the development of K65R-containing minority species may be enhanced during the acute phase of HIV-1 infection, and how this may influence transmitted drug resistance, since a large proportion of onward HIV-1 transmission occurs during this period [77]. Nevertheless, over half of patients who failed therapy with appearance of the K65R mutation were still able to achieve a sustainable virologic response when later treated with regimens containing zidovudine or tenofovir [75], [78].

The increased propensity for template-specific dislocation at the K65 site may not be limited to subtype C. Subtypes F2 and H, as well as CRFs 07_BC, 08_BC and 31_BC also contain the same signature nt polymorphisms at codons 64 and 65 as are found in subtype C RT (Table 1). Studies to assess this are currently underway in our laboratory, although the relative scarcity of these subtypes and CRFs [1], and the fact that they may harbor nt polymorphisms that favor the development of TAMs that antagonize the development of K65R [29], [32], [33], [34], may mitigate against development of K65R in such viruses.

Throughout the RT gene, numerous homopolymeric sequences are present that have potential for mutagenesis. For instance, the 102 region of the RT sequence contains a homopolymeric sequence similar to that of the subtype C K65 region. Preliminary data have shown that RT also experiences characteristic pausing at this specific region (data not shown). Because resistance mutations at position 102 of RT are uncommon, it is unlikely that such mutations arise. However, the 103 region of RT is important in resistance to NNRTIs; therefore, one cannot exclude the possibility that an interaction between positions 102 and 103 might occur that favors NNRTI drug resistance. As a result, it is important to consider the mutagenic potential of homopolymeric nt stretches when evaluating drug resistance development.

Although RNA secondary structure may not directly influence K65R development in regard to subtypes B and C, a role for such secondary structure cannot be excluded for other subtypes and CRFs that share less sequence homology or that contain more polymorphisms. As a result, the study of structural particularities of larger segments of *pol* between subtypes may reveal more re resistance in the protease and integrase segments of *pol*. Such comparisons would also be useful in the determination of dissimilarities with regard to resistance development between the different HIV-1 groups and between HIV-1 and HIV-2, since the latter have less nt homology than exists among HIV-1 subtypes. Our findings suggest that the homopolymeric stretch in the (−)ssDNA intermediate template of subtype C HIV-1 can be considered to be a “mutational hot-spot” associated with development of K65R drug resistance [61], [62].

In conclusion, we have shown that template-specific dislocation mutagenesis is the mechanism whereby subtype C HIV-1 viruses preferentially develop the K65R mutation. Dislocation occurs at the same strong pause site that is responsible for occurrence of K65R that immediately follows a homopolymeric stretch of nt. In addition, we showed that the levels of transcripts containing the mutagenic G nt at the middle position of codon 65 were always greater with the subtype C than subtype B template and that this is independent of the subtype-origin of the RT enzyme used. Our findings confirm and extend the notion that drug resistance mutations can develop differentially among HIV-1 subtypes. Such differences can have clinical relevance [79].

## Supporting Information

Figure S1 **Genomic sequence comparison of the entire RT region of the** ***pol*** **gene derived from subtype B and C HIV-1.** (A) Nucleic acid alignment comparing the nucleic acid sequences of subtype B and C HIV-1 RT showing 90% homology. (B) Amino acid alignment of the amino acid sequences of subtype B and C HIV-1 RT showing 93% amino acid homology.
(TIF)

Figure S2 **Evaluation of the dislocation products under subtype C primer/template conditions with incorrect incorporation of dGTP, dCTP or dTTP.** (A) Graphical representation of DNA transcript production with incorrect nt incorporation from a pool of nt containing dGTP, dCTP and dTTP at the site of K65R development. *Left*: DNA synthesis with subtype B RT on the subtype C template. *Right*: DNA synthesis with subtype C RT on the subtype C template. The product of dislocation without realignment (P+1nt product) is depicted as empty circles and the products of dislocation with realignment or misincorporation without dislocation (P+2nt product) are depicted as empty triangles. During the process of incorrect incorporation of nt, the amount of P+1nt product reaches a maximum at 60 min whereas the amount of P+2nt product continues to rise steadily beyond 60 min. (B) Schematic of dislocation without realignment (P+1nt) and dislocation with realignment or misincorporation without dislocation (P+2nt) products formed as a result of dGTP incorporation at the site of K65R development. The sequence of the unextended primer is depicted in bold.
(TIF)

Figure S3 **K65K, K65T and K65I production as a result of DNA synthesis with primers containing the other three nt possibilities and their impact on dislocation.** (A) Lanes 1 through 10 depict (+)dsDNA synthesis from the (-)ssDNA intermediate with subtype C RT on the subtype B template. The full-length product is observed as a single band at the FL position without dislocation. Lanes 11 through 20 depict (+)dsDNA synthesis from the (-)ssDNA intermediate with subtype C RT on the subtype C template. When A is present at the 3′-end of the primer strand, it correctly binds with the T of the template strand to yield wild-type K65K-containing transcripts. (B) Lanes 1 through 10 depict (+)dsDNA synthesis from the (-)ssDNA intermediate with subtype C RT on the subtype B template. The full-length product is observed as a single band at the FL position with no evidence of dislocation. Lanes 11 through 20 depict (+)dsDNA synthesis from the (-)ssDNA intermediate with subtype C RT on the subtype C template. The C at the 3′-end of the primer strand incorrectly binds with the T of the template strand to yield K65T. (C) Lanes 1 through 10 depict (+)dsDNA synthesis from the (-)ssDNA intermediate with subtype C RT on the subtype B template. No dislocation is present and the full-length product is observed as a single band at the FL position. Lanes 11 through 20 depict (+)dsDNA synthesis from the (-)ssDNA intermediate with subtype C RT on the subtype C template. The T at the 3′-end of the primer strand incorrectly binds with the T of the template strand to yield K65I. Dislocation products are only observed when G is included on the primer strand thereby yielding K65R-containing transcripts.
(TIF)

Figure S4 **Prediction of RNA secondary structure and analysis of the K65 region of RT of subtype B or C HIV-1 or SIV-cpz.** (A) Predicted RNA secondary structure of a part of the subtype B RT segment of *pol*. Codons 64, 65 and 66 are highlighted and the arrow indicates the nt site responsible for the K65R mutation. The subtype B HIV-1 secondary structure of codons 62 through 67 corresponds exactly with published RNA secondary structure data. The Gibbs free energy value for this structure is -65.7 kcal/mole and the K65 codon is located in the unpaired loop of the stem-loop structure. (B) Predicted RNA secondary structure of a part of the subtype C HIV-1 RT segment of *pol*. Codons 64, 65 and 66 are highlighted and the arrow indicates the nt site responsible for the K65R mutation. *Left*: the Gibbs free energy value for the most stable predicted structure is -64.6 kcal/mole and the K65 codon is located in the unpaired loop of the stem-loop structure. *Right*: the Gibbs free energy value for the most second-most stable predicted structure is -63.4 kcal/mole and the K65 codon is located in an unpaired bulge structure. (C) Predicted RNA secondary structure of a part of the SIV-cpz RT segment of *pol*. Codons 64, 65 and 66 are highlighted and the arrow indicates the nt site responsible for the K65R mutation. *Left*: the Gibbs free energy value for the most stable predicted structure is -66.6 kcal/mole and the K65 codon is located between an unpaired loop and a paired stem structure. *Right*: the Gibbs free energy value for the most second-most stable predicted structure is -65.7 kcal/mole and the K65 codon is located in a paired stem structure.
(TIF)
